# Supplementary material for: Iridium-Based Nanohybrids: Synthesis, Characterization, Optical Limiting, and Nonlinear Optical Properties
Source: Nanomaterials (Basel). 2023 Jul 22;13(14):2131. doi: 10.3390/nano13142131 (PMC10385205; doi:10.3390/nano13142131)
Supplement: Supplementary file 1 [file nanomaterials-13-02131-s001.zip › nanomaterials-2508792-supplementary.pdf]

Supporting Information

Iridium-Based Nanohybrids: Synthesis, Characterization, Optical Limiting, and Nonlinear Optical Properties

Nikolaos Chazapis [1,2], Michalis Stavrou [1,2], Georgia Papaparaskeva [3], Alexander Bunge [4], Rodica Turcu [4], Theodora Krasia-Christoforou [3,*] and Stelios Couris [1,2,*]

[1] Department of Physics, University of Patras, 26504 Patras, Greece; n.chazapis@iceht.forth.gr (N.C.); m.stavrou@iceht.forth.gr (M.S.)
[2] Institute of Chemical Engineering Sciences (ICE-HT), Foundation for Research and Technology-Hellas (FORTH), 26504 Patras, Greece
[3] Department of Mechanical and Manufacturing Engineering, University of Cyprus, 1 Panepistimiou Avenue, 2109, Aglantzia, Nicosia, Cyprus; papaparaskeva.georgia@ucy.ac.cy
[4] National Institute R&D of Isotopic and Molecular Technologies, 400293 Cluj-Napoca, Romania;
alexander.bunge@itim-cj.ro (A.B.); rodica.turcu@itim-cj.ro (R.T.)
* Correspondence: krasia.theodora@ucy.ac.cy (T.K.-C.); couris@upatras.gr (S.C.)

Tauc plots

The optical band gap energy, E_g, of the PVP-IrO_2 system was estimated by extrapolating the linear part of curve $(\alpha_0 hv)^2$ with the incident energy (hv) to the x-axis at $(\alpha_0 hv)^2 = 0$, according to Tauc equation [S1]:

$$\alpha_0 hv = C(hv - E_g)^n$$

where h is the Planck's constant, v is the photon's frequency, C is a constant and n=1/2 for direct allowed transitions.

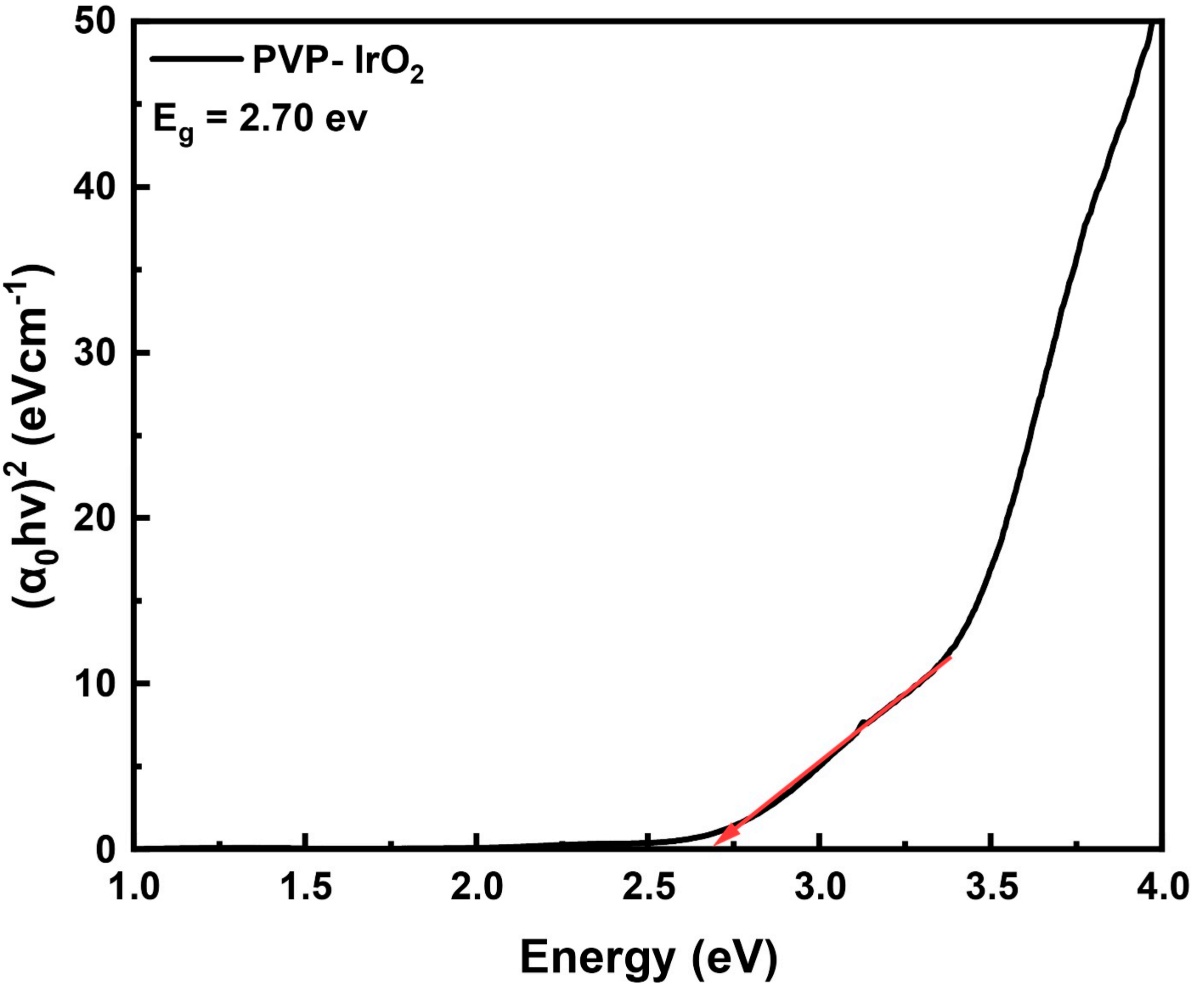

Figure S1. Tauc plots of PVP–IrO_2 for direct optical transitions.

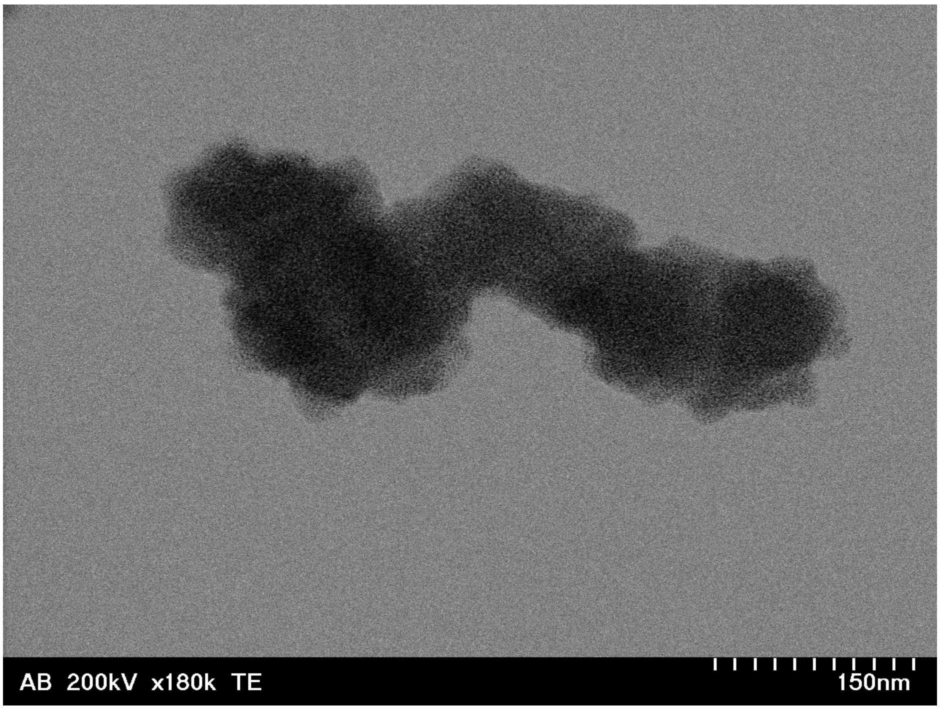

Figure S2. TEM image of sample I (Ir/IrO_2).

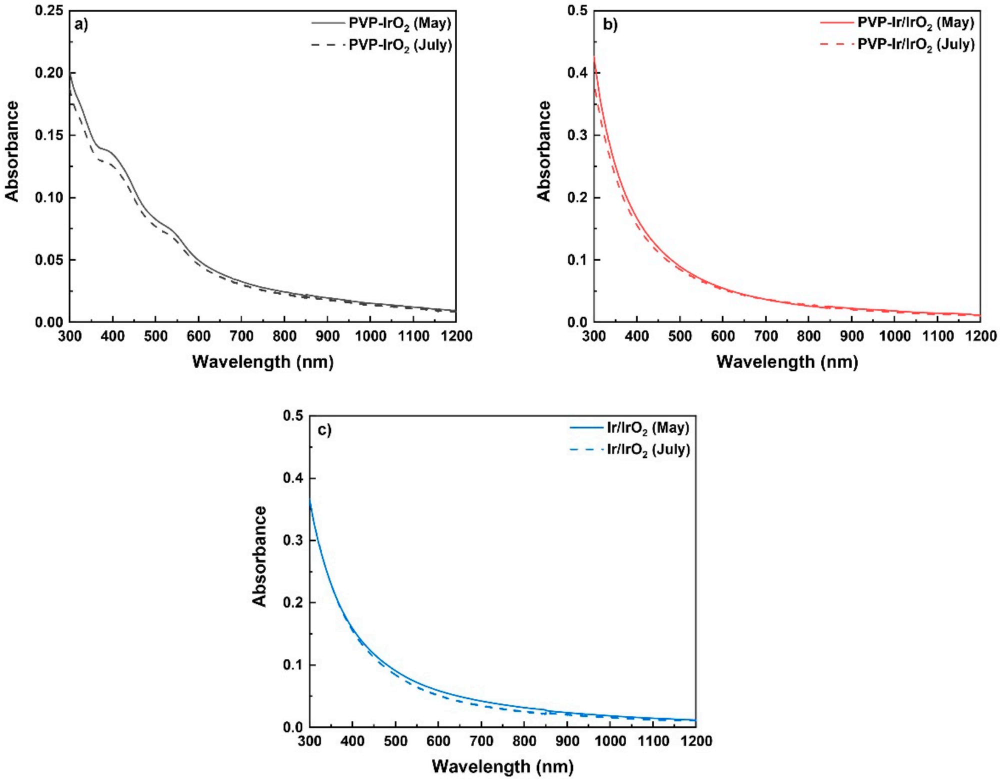

Figure S3: UV-Vis-NIR absorption spectra of (a) PVP-IrO_2, (b) PVP-Ir/IrO_2 and (c) Ir/IrO_2 nanohybrid solutions in different periods.

Table S1. NLO refraction of PVP-IrO_2, PVP-Ir/IrO_2, Ir/IrO_2, Au, ZnO, γ-Fe_2O_3, poly(sodium-4-styrenesulfonate) (PSS)-$Cu(OH)_2$, PSS-$Cu(OH)_2$/CuO and PVP/Pd nanoparticles.

Sample	Excitation conditions	α_0 (cm^{-1})	γ' ($\times 10^{-21}$ m^2/W)	Re$\chi^{(3)}$ ($\times$ 10^{-16} esu)	Re$\chi^{(3)}/\alpha_0$ ($\times$ 10^{-16}) esu cm	Ref.
PVP-IrO_2	4 ns, 532 nm	1.77	-89.8	- 100.5	59.1	this work
PVP-Ir/IrO_2	4 ns, 532 nm	1.8	-20.0	-22.4	12.4	
Ir/IrO_2	4 ns, 532 nm	1.79	-19.9	-22.3	12.4	
PVP-IrO_2	4 ns, 1064 nm	0.39	-28 ± 4	31.9 ± 5.0	82.9 ± 12.0	
PVP-Ir/IrO_2	4 ns, 1064 nm	0.38	-7.8 ± 0.4	8.7 ± 0.4	22.6 ± 1.0	
Ir/IrO_2	4 ns, 1064 nm	0.43	-13.3 ± 2.0	14.7 ± 2.0	34.0 ± 4.0	
PVP/Pd	4 ns, 532 nm	7.5	-238	-330.5	44.1	S2
	4 ns, 1064 nm	6.8	-109	-151.4	22.3	
PSS-$Cu(OH)_2$	4 ns, 532 nm	1	-121	-136	136	S3
PSS-$Cu(OH)_2$/CuO	4 ns, 532 nm	6.8	-436	-489	-489	
γ-Fe_2O_3	4 ns, 532 nm	~5.1	N/A	-9.1	1.8	S4
	4 ns, 1064 nm	~1.15	N/A	3.7	3.2	
Au	4 ns, 532 nm	~0.9	-30.1	-33.7	37.4	S5
ZnO	10 ns, 532 nm	~N/A	120	530	N/A	S6

References

1. Tauc, J. Optical Properties and Electronic Structure of Amorphous Ge and Si. *Mater. Res. Bull.* **1968**, *3*, 37–46.
2. Papagiannouli, I.; Potamianos, D.; Krasia-Christoforou, T.; Couris, S. Third-Order Optical Nonlinearities of PVP/Pd Nanohybrids. *Opt. Mater.* **2017**, *72*, 226–232.
3. Stavrou, M.; Papaparaskeva, G.; Stathis, A.; Stylianou, A.; Turcu, R.; Krasia-Christoforou, T.; Couris, S. Synthesis, Characterization and Nonlinear Optical Response of Polyelectrolyte-Stabilized Copper Hydroxide and Copper Oxide Colloidal Nanohybrids. *Opt. Mater.* **2021**, *119*, 111329
4. Chatzikyriakos, G.; Iliopoulos, K.; Bakandritsos, A.; Couris, S. Nonlinear Optical Properties of Aqueous Dispersions of Ferromagnetic γ-Fe_2O_3 Nanoparticles. *Chem. Phys. Lett.* **2010**, *493*, 314–318
5. Papagiannouli, I.; Aloukos, P.; Rioux, D.; Meunier, M.; Couris, S. Effect of the Composition on the Nonlinear Optical Response of $Au_x Ag_{1-x}$ Nano-Alloys. *J. Phys. Chem. C* **2015**, *119*, 6861–6872
6. . Abrinaei, F.; Molahasani, N. Effects of Mn Doping on the Structural, Linear, and Nonlinear Optical Properties of ZnO Nanoparticles. *J. Opt. Soc. Am. B* **2018**, *35*, 2015
